# Allelic spectrum of formiminotransferase‐cyclodeaminase gene variants in individuals with formiminoglutamic aciduria

**DOI:** 10.1002/mgg3.333

**Published:** 2017-09-11

**Authors:** Ramanath Majumdar, Andrew Yori, Peggy W. Rush, Kimiyo Raymond, Dimitar Gavrilov, Silvia Tortorelli, Dietrich Matern, Piero Rinaldo, Gerald L. Feldman, Devin Oglesbee

**Affiliations:** ^1^ Mayo Clinic College of Medicine Rochester Minnesota; ^2^ Children's Hospital of Michigan Detroit Michigan; ^3^ Wayne State University School of Medicine Detroit Michigan

**Keywords:** Formiminoglutamic acid, formiminotransferase‐cyclodeaminase, inborn errors of metabolism

## Abstract

**Background:**

Elevated plasma and urine formiminoglutamic acid (FIGLU) levels are commonly indicative of formiminoglutamic aciduria (OMIM #229100), a poorly understood autosomal recessive disorder of histidine and folate metabolism, resulting from formiminotransferase‐cyclodeaminase (FTCD) deficiency, a bifunctional enzyme encoded by *FTCD*.

**Methods:**

In order to further understanding about the molecular alterations that contribute to FIGLU‐uria, we sequenced *FTCD* in 20 individuals with putative FTCD deficiency and varying laboratory findings, including increased FIGLU excretion.

**Results:**

Individuals tested had biallelic loss‐of‐function variants in protein‐coding regions of *FTCD*. The *FTCD* allelic spectrum comprised of 12 distinct variants including 5 missense alterations that replace conserved amino acid residues (c.223A>C, c.266A>G, c.319T>C, c.430G>A, c.514G>T), an in‐frame deletion (c.1373_1375delTGG), with the remaining alterations predicted to affect mRNA processing/stability. These included two frameshift variants (c.990dup, c.1366dup) and four nonsense variants (c.337C>T, c.451A>T, c.763C>T, c.1607T>A).

**Conclusion:**

We observed additional *FTCD* alleles leading to urinary FIGLU elevations, and thus, providing molecular evidence of FTCD deficiency in cases identified by newborn screening or clinical biochemical genetic laboratory testing.

## Clinical Report

Formiminoglutamic aciduria (OMIM #229100) is associated with an autosomal recessive condition affecting folate and histidine metabolism and often caused by formiminotransferase‐cyclodeaminase (FTCD, EC 2.1.2.5 to EC 4.3.1.4) deficiency, which is encoded by *FTCD* (HGNC: 3974; OMIM: 606806) (Watkins and Rosenblatt 2014). FTCD is a bifunctional enzyme composed of a formiminotransferase (FT) and cyclodeaminase (CD) domain connected by a short peptide linker and is expressed in liver. The FT domain transfers a formimino group from formiminoglutamate to the N5 position of tetrahydrofolate to generate glutamic acid and 5‐formiminotetrahydrofolate, while the CD domain catalyzes the cyclodeamination of formimino groups yielding N‐5,10 methenyltetrahydrofolate and ammonia (Hilton et al. 2003). FTCD deficiency is described as a clinical spectrum that ranges from benign to severe, but with both extremes associated with developmental delay (Niederwieser et al. 1976). However, the severe form of this disorder was also reported to present with anemia (Arakawa et al. 1965, 1968). It was proposed that the mild end of the spectrum is associated with reduced FT activity, while the severe form results from impeded CD activity, but this hypothesis is disputable (Hilton et al. 2003). Even though *FTCD* was cloned more than 16 years ago (Solans et al. 2000), only three alterations have been reported to cause FTCD deficiency (Hilton et al. 2003) and relatively few studies have addressed any genotype–phenotype relationships to help explain the reported spectrum of this disorder. Thus, we characterized *FTCD* variants in formiminoglutamic aciduric individuals suspected to be FTCD deficient and sought to increase the known number of *FTCD* alleles associated with formiminoglutamic aciduria.

Newborn screening laboratories, using butylation derivatization methods to measure acylcarnitines in dried blood spots by tandem mass spectrometry, have mistaken elevations of formiminoglutamic acid (FIGLU) with elevations of butyryl/isobutyryl‐acylcarnitine (C4‐acylcarnitine) (Malvagia et al. 2006). This is due to the fact that butylated FIGLU is observed to fragment with a positive 287.2 → 85.2 *m/z* ion transition that is difficult to resolve from the ion transition (288.2 → 85.2 *m/z*) that is observed for C4‐acylcarnitines isomers. A C4‐acylcarnitine elevation is a signature for either short‐chain acyl‐CoA dehydrogenase deficiency or isobutyryl‐CoA dehydrogenase deficiency (Oglesbee et al. 2007).

## Ethical Compliance

This study was approved by the Mayo Clinic's Institutional Review Board.

A total of 22 individuals (20 cases and 2 parents of 2 participants) from 17 unrelated families were found either to have FIGLU‐uria or to be related to an individual with this condition. The diagnosis of most of the cases was based on elevated FIGLU levels determined in dried blood spots by tandem mass spectrometry (Malvagia et al. 2006). In addition, we quantified urinary FIGLU levels for a handful of positive cases (Table 1). Cases provided whole blood or residual dried blood spots for isolating DNA and *FTCD* sequencing. Cases were reported to have trans‐continental ancestry, including Europe, Asia, Middle East, and Africa, indicating that this is a pan‐ethnic condition.

**Table 1:** Formiminotransferase‐cyclodeaminase (FTCD) variant spectrum

Case ID	Urine FIGLU (normal: undetected; mmol/mol Cr)	*FTCD* genotype			
		Variant 1	AF (ExAC)	Variant 2	AF (ExAC)
Case #1	NA	c.990dup; p.Pro331Alafs*2	0.002	c.990dup; p.Pro331Alafs*2	0.002
Case #2	NA	c.990dup; p.Pro331Alafs*2	0.002	c.223A>C; p.Met75Leu	NR
Case #3	NA	c.990dup; p.Pro331Alafs*2	0.002	c.990dup; p.Pro331Alafs*2	0.002
Case #4	NA	c.990dup; p.Pro331Alafs*2	0.002	c.763C>T; p.Arg255*	0.00003
Case #5	NA	c.1373_1375delTGG; p.Val458del	NR	c.1607T>A; p.Leu536*	0.0004
Case #6	NA	c.990dup; p.Pro331Alafs*2	0.002	c.990dup; p.Pro331Alafs*2	0.002
Case #7	NA	c.990dup; p.Pro331Alafs*2	0.002	c.990dup; p.Pro331Alafs*2	0.002
Case #8	NA	c.990dup; p.Pro331Alafs*2	0.002	c.1607T>A; p.Leu536*	0.0004
Case #9	195	c.990dup; p.Pro331Alafs*2	0.002	c.514G>T; p.Gly172Trp	NR
Case #10	54	c.319T>C, p.Cys107Arg	0.0002	c.451A>T; p.Lys151*	NR
Case #11	NA	c.990dup; p.Pro331Alafs*2	0.002	c.430G>A; p.Gly144Arg	0.0002
Case #12	33	c.990dup; p.Pro331Alafs*2	0.002	c.763C>T; p.Arg255*	0.000008
Case #13	NA	c.990dup; p.Pro331Alafs*2	0.002	c.266A>G; p.Asp89Gly	NR
Case #14	11	c.990dup; p.Pro331Alafs*2	0.002	c.1366dup; p.Glu456Glyfs*56	0.0001
Case #15	5	c.990dup; p.Pro331Alafs*2	0.002	c.1366dup; p.Glu456Glyfs*56	0.0001
Mother of 14 and 15	NA	c.990dup; p.Pro331Alafs*2	0.002	ND	
Father of 14 and 15	NA	c.1366dup; p.Glu456Glyfs*56	0.0001	ND	
Case #16	20	c.990dup; p.Pro331Alafs*2	0.002	c.223A>C; p.Met75Leu	NR
Case #17	67	c.990dup; p.Pro331Alafs*2	0.002	c.1607T>A; p.Leu536*	0.0004
Case #18	61	c.990dup; p.Pro331Alafs*2	0.002	c.1607T>A; p.Leu536*	0.0004
Case #19	80	c.990dup; p.Pro331Alafs*2	0.002	c.337C>T; p.Gln113*	NR
Case #20	84	c.990dup; p.Pro331Alafs*2	0.002	c.319T>C; p.Cys107Arg	0.0002

Biallelic variants of *FTCD* and consequence to functional protein are depicted under *FTCD* genotype. Elevated FIGLU level were indicated by pseudoelevations of C4‐acylcarnitine when screening acylcarnitines in dried blood spots by tandem mass spectrometry (see text for details). AF, allele frequency as seen in the ExAC database; *NR, not reported in ExAC; NA, not available.

For *FTCD* sequencing, genomic DNA was extracted from whole blood or dried blood spots using a PureGene extraction protocol (Roche, Indianapolis, IN) or the QIAmp Tissue Kit extraction protocol (Qiagen, Germantown, MD) according to the manufacturer's specifications. PCR was used to simultaneously amplify 14 protein‐encoding exons of *FTCD* (RefSeq NM_206965.1, genome build GRCh37/hg19). PCR primers were designed with 5′ “universal” forward (19 bases not found in the human genome) or reverse (23 bases not found in the human genome) “tags” of sequence that were incorporated into the generated PCR product (Table  S1). These primers were optimized for a universal‐sequencing primer PCR system. The final PCR reaction mixture contained genomic DNA, 2X PCR PreMix Buffer D (Epicentre Biotechnologies, Madison, WI), 1.25 mmol/L MgCl_2_, and 1.25 *μ*mol/L each forward and reverse primer. A touchdown PCR method (a hot‐start of 3 min at 95°C, followed by 15 cycle of denaturation at 95°C for 30 sec, annealing 65°C for 30 sec, which was decreased by 0.5°C each subsequent cycle, and extension at 72°C for 60 sec, followed by another 16 cycles of denaturation at 95°C for 30 sec, annealing 58°C for 30 sec, and extension at 72°C for 60 sec followed by a final extension of 72°C for 10 min) on a Veriti™ Thermal Cycler (Applied Biosystems, Foster City, CA). Amplified PCR products were purified with an Agencourt^®^ AMPure XP Purification Kit (Beckman Coulter, Indianapolis, IN), sequenced in both direction using universal primers and a BigDye Terminator™ v1.1 cycle sequencing kit (Applied Biosystems), further purified using the Agencourt^®^ CleanSEQ Dye‐Terminator Removal Kit (Beckman Coulter). The purified sequencing products were loaded onto the ABI 3730xl DNA Analyzer capillary electrophoresis instrument. DNA sequencing data were managed through GeneSifter software (Geospiza, Seattle, WA). Sequencing data files were analyzed using the Variant Surveyor™ v3.24 (SoftGenetics, State College, PA). No pseudogenes or other areas with the potential for coamplification were found with individual primers via a BLAT search (Dreszer et al. 2012). In addition, genomic areas, to which primers were predicted to bind, were free of reported SNPs according to SNPCheck v.3.0 (https://secure.ngrl.org.uk/SNPCheck/snpcheck.htm). All novel variations detected in this study were verified to be unique by a search with dbSNP (Sherry et al. 2001) and the Exome Aggregation Consortium (ExAC) genome browser (Lek et al. 2016) was queried to determine putative minor allele frequencies. Whether *FTCD* sequence variants were neutral polymorphisms or novel alterations with putative functional consequences was further addressed by (1) screening 100 alleles from healthy control subjects for each alteration, (2) employing conventional annotation tools including MutationTaster2 (Schwarz et al. 2014), MutPred (Li et al. 2009), SIFT (Vaser et al. 2016), Polyphen2 (Adzhubei et al. 2010), and (3) by comparing evolutionary conservation of amino acid residues across different species.

Elevated FIGLU levels were observed by tandem mass spectrometry in urine from cases as a part of follow‐up of an abnormal newborn screening result and prior to molecular characterization (Table 1). Urinary FIGLU concentrations ranged from 5 to 195 mmol/mol creatinine (normal: not detected). Twenty of 22 tested individuals were found to have biallelic alterations in protein‐coding regions of *FTCD* (Table 1). Two other individuals were obligate carriers for *FTCD* variants as they were the parents of two cases. The *FTCD* variant profile was highly heterogeneous and 10 distinct previously unreported genotypes were detected in this study. Of note, 23 of the total 42 alterations were due to a common c.990dup frameshift alteration (p.Pro331Alafs*2), four individuals were found to be homozygous for this c.990dup variant, the remainder were rare compound heterozygotes for other alleles.

Among this latter group, 14 individuals harbored c.990dup *in trans* to a single nonsense variant (c.763C>T, p.Arg255*; c.1607T>A, p.Leu536*; c.451A>T, p.Lys151*; c.337C>T, p.Gln113*), an in‐frame deletion (c.1373_1375delTGG, p.Val458del), a frameshift variant (c.1366dup, p.Glu456Glyfs*56), or a missense variant (c.223A>C, p.Met75Leu; c.266A>G, p.Asp89Gly, c.319T>C, p.Cys107Arg; c.430G>A, p.Gly144Arg; c.514G>T, p.Gly172Trp). Two cases, who were devoid of c.990dup, were compound heterozygous for another alteration (p.Lys151*, p.Cys107Arg; p.Leu536*, or p.Val458del). Aside from five novel missense variants, all of the newly described variants were expected to either affect mRNA processing, lead to protein truncation, or remove a highly conserved amino acid residue.

**Figure 1:**
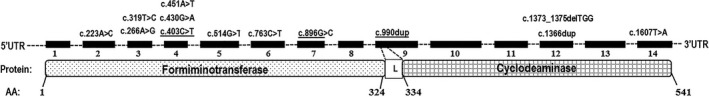
The distribution of formiminotransferase‐cyclodeaminase (FTCD) variants in relation to protein functional domains and exonic location. The illustration of FTCD positions for 14 exons (filled boxes numbered 1–14) and associated introns (broken lines). Bottom panel represents the location of the FT and CD protein subunits (formiminotransferase [amino acids, 1–324], cyclodeaminase [amino acids, 334–541], and the linker [L] [amino acids, 325–333]). AA indicates amino acids in relation to FTCD peptide sequence.

Figure 1 shows the location of each alteration detected in relation to *FTCD* and its protein product. In addition to the putative pathological variants, a total of 24 *FTCD* polymorphisms with high minor allele frequencies were identified within exons, introns, or the 5′ untranslated region. The functional relevance of the novel *FTCD* missense variants and polymorphisms was investigated using the MutationTaster, MutPred, PolyPhen2, and SIFT analysis tools. To assess the predictive power of this bioinformatic approach, the analysis was performed on both newly identified missense variants and previously reported missense variants (Tables 1 and 2). All in silico prediction tools successfully classified previously known missense alterations (Hilton et al. 2003). Further investigation of the five missense variants (p.Met75Leu, p.Asp89Gly, p.Cys107Arg, p.Gly144Arg, p.Gly172Trp) identified in this study indicated all five to have a high probability of being deleterious (Table 2). Additional support for the pathological significance of the missense variants identified in the present study came from the analysis of evolutionary conservation of the mutated residues in nine orthologous (vertebrate) FTCD proteins and by comparing the reported minor allele frequency for variants listed in the ExAC database, an online resource of gene variants from 60,706 subjects. All 10 novel variants had an average allele frequency of <0.0004 and were not detected in 100 normal subjects tested in the laboratory, but were predicted to lead to protein truncation (Table 1).

**Table 2:** In silico pathogenicity scores for *FTCD* variants

Variants	MutationTaster		MutPred		Polyphen2		SIFT	
	Score	Consequence	Score	Consequence	Score	Consequence	Score	Consequence
c.223A>C; p.Met75Leu	1	Disease causing	0.75	Deleterious	0.80	Possibly damaging	0.68	Tolerated
c.266A>G; p.Asp89Gly	1	Disease causing	0.86	Deleterious	1.00	Probably damaging	0.08	Tolerated
c.319T>C, p.Cys107Arg	1	Disease causing	0.86	Deleterious	1.00	Probably damaging	0.01	Deleterious
c.430G>A; p.Gly144Arg	1	Disease causing	0.80	Deleterious	1.00	Probably damaging	0	Deleterious
c.514G>T; p.Gly172Trp	1	Disease causing	0.90	Deleterious	1.00	Probably damaging	0	Deleterious
c.337C>T; p.Gln113*	1	Disease causing	–^$^		–		–	
c.451A>T; p.Lys151*	1	Disease causing	–		–		–	
c.763C>T; p.Arg255*	1	Disease causing	–		–		–	
c.1607T>A; p.Leu536*	1	Disease causing	–		–		–	
c.1373_1375delTGG; p.Val458del	0.99	Disease causing	–		–		–	
c.1366dup; p.Glu456Glyfs*56	1	Disease causing	–		–		–	

The prediction score for probable pathogenicity of the novel *FTCD* novel variants was depicted using conventional in silico tools (see text for details). –^$^, the pathogenicity scores for nonsense, frameshift or deletion mutations were unable to be characterized using some in silico tools.

With our analysis, we found a number of functionally relevant *FTCD* variants, only three of which were previously known (Fig. 1). Ten of these alterations were observed for the first time from this analysis. A common variant, c.990dup, which was previously described (Hilton et al. 2003), was also observed in our cohort. Most of the novel variants described here represent profound alterations, and are ultimately predicted to lead to either premature stop codons, or as for c.990dup, changes to the *FTCD* reading frame that interrupts the peptide linker between the FT and CD domains of the enzyme (Figure 1), and are predicted to give rise to aberrant FTCD. Functional characterization of all novel sequence variants identified in this study would be helpful to confirm their significance and to determine their effect on protein function. Previous studies indicated that higher levels of urine FIGLU are observed in cases with milder forms of the disease and these levels occur in the absence of histidine loading (Hilton et al. 2003), whereas the presence of FIGLU in urine is typically only observed in severe cases only after l‐histidine administration (Arakawa et al. 1965, 1968). Two mutations, p.R135C and p.R299P, associated with a reportedly severe phenotype uncovered by l‐histidine loading were not detected in the present study (Hilton et al. 2003). In addition, the severe form of this condition is believed to be associated with elevated serum folate levels, whereas the milder form of disease is not. In contrast, we found persistent FIGLU excretion in the cases whose urine specimens were tested to date, and surprisingly, serum folates were also normal when tested.

From our experience, we estimate that the incidence of FTCD deficiency is 1 in 46,449 births as determined by population screening with tandem mass spectrometry. This is based on the observation that 15 FIGLU cases were detected among 696,730 births between 2005 and 2011 in the U.S. state of Michigan (G. L. Feldman, pers. comm.). The clinical study of these newborn screen‐positive cases is presently ongoing, but we are aware that our FIGLU cohort contained one patient with mild cognitive delay and another with developmental delay that was most likely due to another etiology. However, clinical follow‐up has yet to identify additional developmental concerns nor was anemia prevalent in this cohort when tested. Thus, it is questionable whether congenital FIGLU‐uria due to FTCD deficiency is a condition of short‐term clinical concern. Indeed, our estimated incidence would indicate that numerous FTCD‐deficient cases are undiagnosed or unrecognized by either current laboratory testing or from the lack of a clinical presentation.

It is evident from this report that variants associated with increased FIGLU excretion are distributed throughout *FTCD* without any preferential clustering within sequences encoding either the FT or CD domain of the mature FTCD protein. Further studies following FTCD deficiency cases with an assessment of their clinical findings will enable a better understanding of the long‐term clinical impact of this inborn error of metabolism.

## Conflict of Interest

The authors have no conflicts to declare.

## Supporting information


**Table S1.** FTCD template PCR primer sequences.
