# Supplementary material for: Allelic spectrum of formiminotransferase‐cyclodeaminase gene variants in individuals with formiminoglutamic aciduria
Source: Mol Genet Genomic Med. 2017 Sep 11;5(6):795–9. doi: 10.1002/mgg3.333 (PMC5702579; doi:10.1002/mgg3.333)
Supplement: Supplementary file 1 — Table S1. FTCD template PCR primer sequences. [file MGG3-5-795-s001.docx]

Supplemental Table 1. FTCD Template PCR Primer Sequences

| Exon | Primers | | Primer Sequence (5’ to 3’) | Product Size |
| --- | --- | --- | --- | --- |
| 1 | Forward | FTCD UPS 1F | *XCCCACTGCTCCTTGTGCCT | 304 bp |
|  | Reverse | FTCD UPS 1R | *ZCCTTAGCCACTCAAGCTGCC |  |
| 2 | Forward | FTCD UPS 2F | XTCAGCCCCCATTTGTGTCTC | 495 bp |
|  | Reverse | FTCD UPS 2R | ZGCCATGACCCCCACACTC |  |
| 3 | Forward | FTCD UPS 3F | XCTTGCTGGGTGTGTGGACT | 377 bp |
|  | Reverse | FTCD UPS 3R | ZAGGGAACTGGGGGATACAGA |  |
| 4 | Forward | FTCD UPS 4F | XTTGCTTGCGGTTCTGTTTATCAT | 599 bp |
|  | Reverse | FTCD UPS 4R | ZTTCGCTCTGGGGTGAGACA |  |
| 5 | Forward | FTCD UPS 5F | XGGTGGACCTGCCCCTTTGT | 586 bp |
|  | Reverse | FTCD UPS 5R | ZCTGCCTCGTTCCCTGGAAAAT |  |
| 6 | Forward | FTCD UPS 6F | XATGCAAGTGCCAGGAGAGAC | 347 bp |
|  | Reverse | FTCD UPS 6R | ZCCTGGCTGGAGGATGTGG |  |
| 7 | Forward | FTCD UPS 7F | XAGCCGCTGCTGCTGTGAG | 367 bp |
|  | Reverse | FTCD UPS 7R | ZGAGCTCCGCCACCGCCTC |  |
| 8 | Forward | FTCD UPS 8F | XAGGGGGCAACTCCTTTCTCTG | 602 bp |
|  | Reverse | FTCD UPS 8R | ZGGTGACCACTCGGCTGAGAAC |  |
| 9 | Forward | FTCD UPS 9F | XGCAGGTTCTCAGCCGAGTGG | 362 bp |
|  | Reverse | FTCD UPS 9R | ZCTATCTTTAAACTACAAATCTAACAACAACTGCGCCTCCCCTG |  |
| 10 | Forward | FTCD UPS 10F | XCTATCTTTAAACTACAAATCTAACTGCGACCCCCACATCCAC | 341 bp |
|  | Reverse | FTCD UPS 10R | ZAGGCTGACCCGCTTCTCACTG |  |
| 11 | Forward | FTCD UPS 11F | XAAACCACTGGCCCAACAGAA | 360 bp |
|  | Reverse | FTCD UPS 11R | ZAGTCATTCCCAAACACCCAGCA |  |
| 12 | Forward | FTCD UPS 12F | XGCTGGGTGTTTGGGAATGAT | 434 bp |
|  | Reverse | FTCD UPS 12R | ZTGCCCGTGAAGTGAGGTCTC |  |
| 13 | Forward | FTCD UPS 13F | XCGCTGGGCTCCTTGTGAG | 415 bp |
|  | Reverse | FTCD UPS 13R | ZGGGAAAGAGGGGTCTGGTAGTT |  |
| 14 | Forward | FTCD UPS 14F | XCTGGGCTGAAGACTGGAACT | 470 bp |
|  | Reverse | FTCD UPS 14R | ZGCCAACACACAACACAGGTT |  |

*X= GGGTTCCCTAAGGGTTGGA (Forward tag); *Z= GTGCCAGCAAGATCCAATCTAGA (Reverse tag). These are universal sequencing primers that are incorporated into the PCR product of each exon. Spacer sequence in FTCD UPS 9R and FTCD UPS 10F primers are underlined. *FTCD* is GenBank Accession: NC_000021.8; Chr 21: 47556065 – 47575499, complement –GRCh37, Ref Seq. NM_206965.1
